# The double edge of anti-CD40 siRNA therapy: It increases renal microcapillar density but favours the generation of an inflammatory milieu in the kidneys of ApoE^**−/−**^ mice

**DOI:** 10.1186/s12950-019-0228-9

**Published:** 2019-12-16

**Authors:** Miguel Hueso, Angela Casas, Adrian Mallén, Laura de Ramón, Nuria Bolaños, Cristian Varela, Josep M. Cruzado, Joan Torras, Estanislao Navarro

**Affiliations:** 1 Department of Nephrology, Hospital Universitari Bellvitge, and Bellvitge Research Institute (IDIBELL). L’Hospitalet de Llobregat, 08907 Barcelona, Spain; 2 Laboratori de Nefrología Experimental, Bellvitge Research Institute (IDIBELL). L’Hospitalet de Llobregat, Barcelona, Spain; 3 Independent Researcher, Barcelona, Spain

**Keywords:** siRNA therapy, Off-target side effects, Inflammatory milieu, Kidney, Atherosclerosis, Macrophage infiltration, Innate immunity

## Abstract

**Background:**

Chronic kidney disease (CKD) is associated with endothelial dysfunctions thus prompting links between microcirculation (MC), inflammation and major cardiovascular risk factors.

**Purpose of the study:**

We have previously reported that siRNA-silencing of CD40 (siCD40) reduced atherosclerosis (ATH) progression. Here, we have deepened on the effects of the siCD40 treatment by evaluating retrospectively, in stored kidneys from the siCD40 treated ApoE^−/−^ mice, the renal microcirculation (measured as the density of peritubular capillaries), macrophage infiltration and NF-κB activation.

**Methods:**

Kidneys were isolated after 16 weeks of treatment with the anti-CD40 siRNA (siCD40), with a scrambled control siRNA (siSC) or with PBS (Veh. group). Renal endothelium, infiltrating macrophages and activated NF-κB in endothelium were identified by immunohistochemistry, while the density of stained peritubular capillaries was quantified by image analysis.

**Results:**

ATH was associated with a reduction in renal MC, an effect reversed by the anti-CD40 siRNA treatment (3.8 ± 2.7% in siCD40; vs. 1.8 ± 0.1% in siSC; or 1.9 ± 1.6% in Veh.; *p* < 0.0001). Furthermore, siCD40 treatment reduced the number of infiltrating macrophages compared to the SC group (14.1 ± 5.9 cells/field in siCD40; vs. 37.1 ± 17.8 cells/field in siSC; and 1.3 ± 1.7 cells/field in Veh.; *p* = 0.001). NF-κB activation also peaked in the siSC group, showing lower levels in the siCD40 and Veh. groups (63 ± 60 positive cells/section in siCD40; vs. 152 ± 44 positive cells/section in siSC; or 26 ± 29 positive cells/section in veh.; *p* = 0.014). Lastly, serum creatinine was also increased in the siCD40 (3.4 ± 3.3 mg/dL) and siSC (4.6 ± 3.0 mg/dL) groups when compared with Veh. (1.1 ± 0.9 mg/dL, *p* = 0.1).

**Conclusions:**

Anti-CD40 siRNA therapy significantly increased the density of peritubular capillaries and decreased renal inflammation in the ATH model. These data provide a physiological basis for the development of renal diseases in patients with ATH. Furthermore, our results also highligth renal off-target effects of the siRNA treatment which are discussed.

**Graphical abstract:**

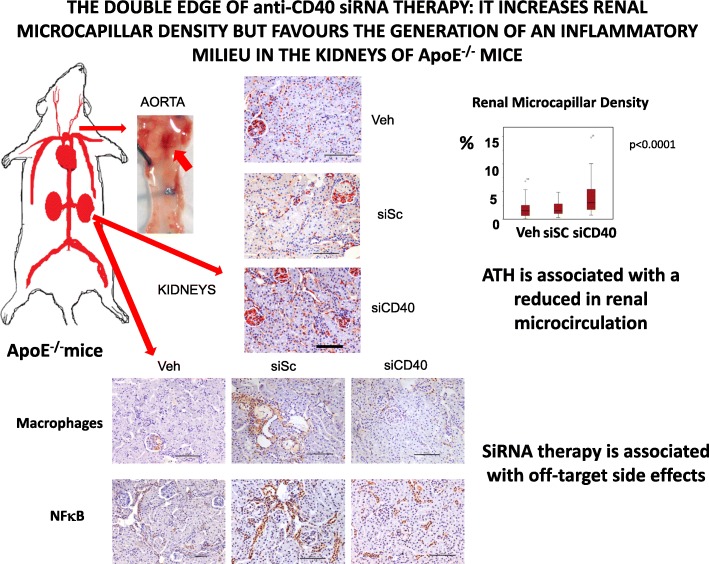

## Background

Atherosclerosis (ATH) is associated with a systemic inflammatory state characterized by the activation of endothelial and blood cells and by an increase in the plasmatic concentration of inflammatory factors [1]. Inflammation is also involved in renal injury and may trigger compensatory mechanisms to offset tissue injury-dependent ATH lesions, suggesting a close interrelation among them [2]. In addition, there are evidences of the existence of a physiological link between renal microcirculation (MC) and ATH progression [3], supporting the relevance of kidney in the pathogenesis of ATH. In this sense, it has been described that kidneys have a key regulatory role in the renin-angiotensing system (RAS) [4], and that the blockade of RAS reduces both the development of cardiovascular and renal diseases [5]. Thus, the activation of inflammatory pathways could promote kidney injury and accelerate the progression of atherosclerotic lesions.

Endothelium is a key regulator of vascular homeostasis, with autocrine and paracrine functions, and it is known that activation of the endothelium in the renal microvascular network cause inflammation and the activation of the Nuclear Factor-κB (NF-κB) pathway [6]. Kidneys are highly sensitive to hypoxia because of their high oxygen demand from tubular cells and the low oxygen tensions present in the medulla [7]. Insults to endothelial-cells (EC) activate oxidative systems, such as the NADPH oxidase complex, that produce hydrogen peroxide which subsequently causes oxidative stress [8]. Furthermore, this stimulates the phosphorylation of NF-κB, which leads to the activation of EC, the upregulation of chemotactic factors and adhesive receptors, and to the recruitment of leukocytes [8]. In this sense, it its known that NF-κB activates many of the genes involved in the inflammatory response network that are pivotal in the pathogenesis of vascular injury [9], and that activated NF-κB could have a role in the progression of kidney diseases [10], thus suggesting that NF-κB activation could be the physiological link among vascular injury and the development of renal diseases.

CD40/CD40L interaction has a critical role in ATH pathogenesis since this stimulates the NF-κB pathway and upregulates the expression of proinflamatory and proatherogenic genes [11]. Endothelial cell expression of CD40 is increased by acute and chronic inflammatory stimuli [12], and CD40/CD40L signalling has been implicated in inflammation-enhanced microvascular thrombosis [13]. On the other hand, it has been reported that ablation of CD40, either by silencing with an anti-CD40-specific siRNA or by genetic deletion (as CD40^−/−^ ApoE^−/−^ double knockout mice), caused a significant reduction in the area of atherosclerotic plaques, also associated with a decrease of inflammation [14, 15].

The renal effects of the siCD40 treatment have been tested by our group in rat models of renal transplantation and in experimental models of Lupus Nephritis, which showed an improvement in the renal function in a dose dependent manner as well as the preservation of the renal architecture [16, 17]. Nevertheless, a number of concerns have been raised on the off-target side effects of siRNA therapies likely caused by the activation of the innate immune system through the RNA-sensing pattern recognition (PRRS) of siRNAs [18]. Here, we deepened on the functional relationships among kidneys and aorta by studying the renal microvascular network and the degree of renal inflammation in ApoE^−/−^ mice treated with the siCD40 therapy. We have also addressed the off-target side effects of siRNA therapies, and here we show that this resulted in a significant increase in renal inflammation in the ApoE^−/−^ mice, with the subsequent impact on renal function.

## Results

### Anti-CD40 siRNA treatment increased microcapillar density in the kidneys of treated mice

**Fig. 1:**
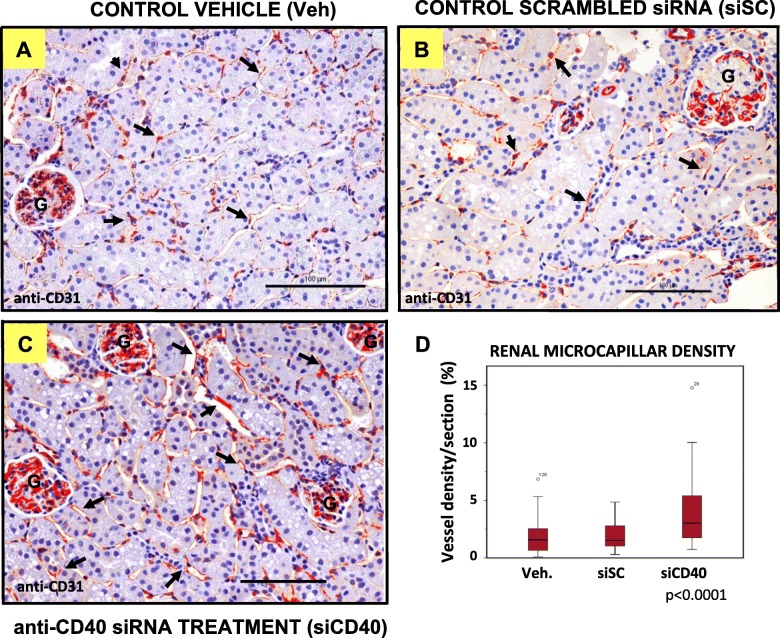
Anti-CD40 siRNA treatment increases microcapillar density in kidneys from ApoE-deficient mice. Representative images of the anti-CD31(PCAM-1)/haematoxylin staining of kidney sections from ApoE^−/−^ mice treated with: **a** control vehicle (Veh), **b** a control sequence-scrambled siRNA (siSC), or **c** the anti-CD40 siRNA (siCD40), at 200x magnification (Scale bars are 100 µm). To increase contrast and facilitate visualization, endothelial cells (EC) were stained in false red with ImageJ. G shows the renal glomeruli and the arrows some EC. **d** Box-plot showing the proportion of vessel area density per kidney section (see Material and Methods for details on the measurement). Each diagram represents the median, quartiles and outliers. The *colored box* represents the interquartile range that contains 50% of the values. The whiskers are lines that extended from the box to the highest and lowest values, excluding outliers. A line across the box represents the median value. Kruskal Wallis test

We have previously shown that silencing of the costimulatory receptor CD40 with a specific siRNA (siCD40 group) ameliorated progression of experimental ATH in the ApoE^−/−^ model mouse when compared with control animals treated with a scrambled-sequence siRNA (siSC group) or with vehicle (Veh. group) [15]. We hypothesized that ATH progression could be also associated to a reduction in renal microcirculation, and here we show that the siCD40 therapy impacted on the renal microcapillar density, measured as the number of PECAM-1 positive cells per optical field at a 200x magnification. As can be seen in Fig. 1a–d, the endpoint of ATH progression after 16 weeks of treatment showed similar values for PECAM-1 positive cells in the two control groups, treated with vehicle only (1.9 ± 1.6% of positive cells, *n* = 10 mice) or with the siSC (1.8 ± 0.1% of positive cells, *n* = 5), while the value for the mice treated with the siCD40 was significantly higher (3.8 ± 2.7% of positive cells, n = 10; *p* < 0.0001).

### SiCD40 treatment increased macrophage infiltration and NF-κB activation in the kidneys of treated mice

**Fig. 2:**
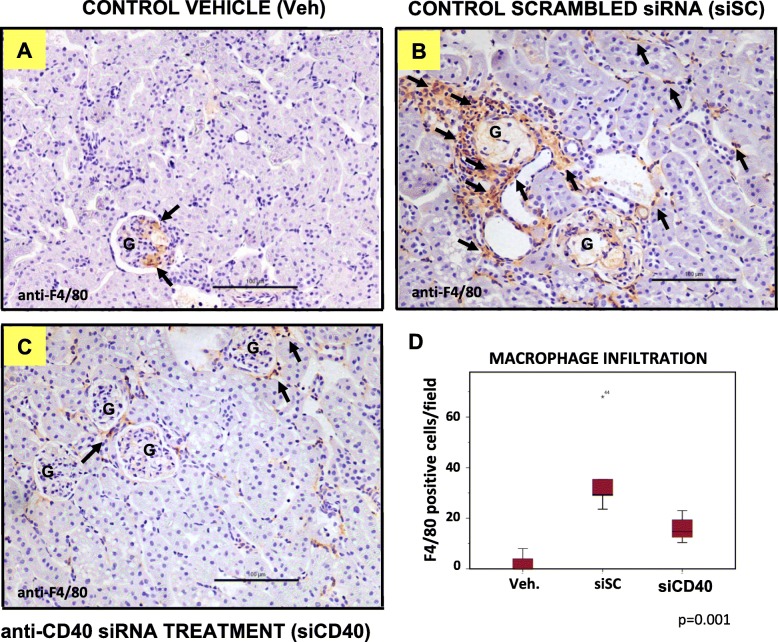
Anti-CD40 siRNA treatment increases macrophage infiltration in the kidneys of treated mice. Representative images of F4-80/haematoxylin staining of kidney sections from ApoE^−/−^ mice treated with: **a** control vehicle (veh), **b** a control sequence-scrambled siRNA (siSC), or **c** the anti-CD40 siRNA (siCD40), at 200x magnification (Scale bars are100 μm). G shows the renal glomeruli and a number of positive cells are marked by arrows. **d** Box-plot showing the number of F4/80+ cells per kidney section. Each diagram represents the median, quartiles and outliers. The *colored box* represents the interquartile range that contains 50% of the values. The whiskers are lines that extended from the box to the highest and lowest values, excluding outliers. A line across the box represents the median value. Kruskal Wallis test

In our previous work, we described a macrophage miRNA/mRNA signature for ATH progression in siCD40 treated mice [15]. Since siRNA duplexes are known to be potent activators of the innate immune system [19] we aimed to study possible “off-target” effects of the therapy by focusing in macrophage and inflammatory responses. To achieve this objective, we measured macrophage infiltration (as F4/80-positive cells) and NF-κB activation (as NF-κB-p65 positive nuclear staining) by IHC in sections of the kidneys of mice treated with the control vehicle, the control siSC or with the siCD40 for 16 weeks. Figure 2a–d clearly shows that kidneys from mice treated with the two siRNAs (siSC and siCD40) showed higher values of infiltrating F4/80-positive cells when compared with the vehicle control. Mice from the siSC group showed the highest levels of macrophage infiltration, 37.1 ± 17.8 F4/80 positive cells/section (Fig. 2b, d) vs.1.2 ± 1.7% F4/80 positive cells/section for animals treated with the vehicle only (Fig. 2a, d). Macrophages were predominantly located in tubular capillaries and around the glomeruli in the siSC group, and glomerular lesions were characterized by a foam cell appearance (Fig. 2b). Treatment with the siCD40 partially reversed the increase seen in the SC group (14.1 ± 5.9 F4/80 positive cells/section; *p* = 0.001), but did not recover the vehicle control values (Fig. 2c, d).

**Fig. 3:**
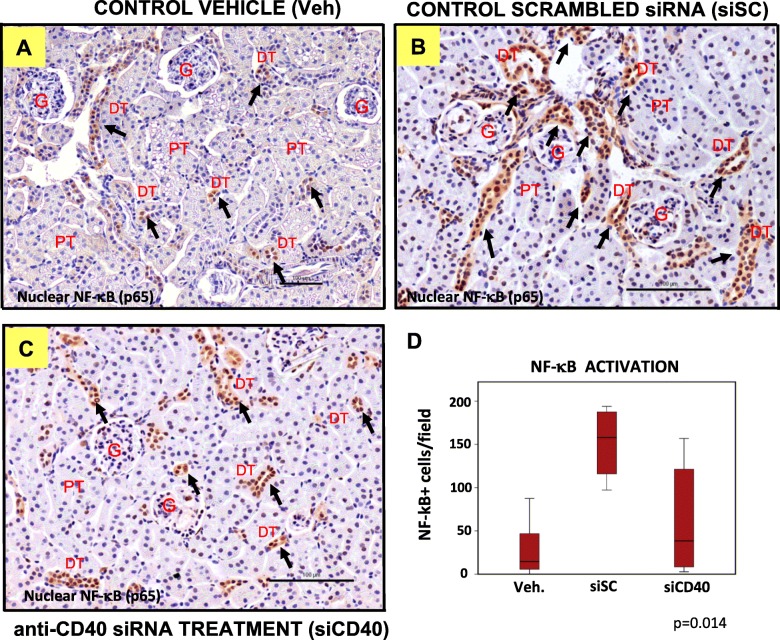
Anti-CD40 siRNA treatment increases NF-κB activation in the kidneys of treated mice. NF-κB-p65 Representative images of NF-κB-p65/haematoxylin staining of kidney sections from ApoE^−/−^ mice treated with: **a** control vehicle (veh), **b** a control sequence-scrambled siRNA (siSC), or **c** the anti-CD40 siRNA (siCD40), at 200x magnification (Scale bars are100 μm). A number of positive cells are marked by arrows. G shows the renal glomeruli, PT shows some proximal tubules characterized by large cells with an apical brush border (not all nuclei are visible in the cross-section) and DT shows some distal tubules with smaller cells. **d** Box-plot showing the number of NF-κB-p65 + cells per kidney section. Each diagram represents the median, quartiles and outliers. The *colored box* represents the interquartile range that contains 50% of the values. The whiskers are lines that extended from the box to the highest and lowest values, excluding outliers. A line across the box represents the median value. Kruskal Wallis test

On the other hand, activation of NF-κB has been associated to the progression of renal tubulointerstiticial lesions in experimental proteinuric nephropathies and in the development of glomerulonephritis [10]. Following to macrophage infiltration, we measured NF-κB activation (by counting nuclear NF-κB-p65-positive cells) in the same experimental model (Fig. 3a–d). Similarly, to the effect described for F4/80-positive cells, nuclear NF-κB-p65 peaked in the siSC group, with values of 152 ± 44 positive cells/section (Fig. 3b, d), when compared values measured in the control group treated with the vehicle only which showed 26 ± 29 positive cells/section (Fig. 3a, d). Enhanced NF-κB-p65 activation was clearly detected in tubules, EC and inflammatory infiltrated cells in the siSC group (Fig. 3b), and as for macrophage infiltration, the effect of NF-κB activation was partially reversed by the treatment with the siCD40 up to 63 ± 60 positive cells/section (*p* = 0.014), although in this case the inter-individual variability was very high (Fig. 3c, d).

Lastly, we also measured serum creatinine as a measure of renal function. The two groups treated with siRNAs (siCD40 and siSC) showed increased levels of serum creatinine when compared with the vehicle control group, (3.4 ± 3.3 mg/dL in the siCD40 treated group, *n* = 10; 4.6 ± 3.0 mg/dL in the siSC group, *n* = 5; 1.1 ± 0.9 mg/dL in vehicle group, n = 10; *p* = 0.1), suggesting the existence of off-target side renal effects. On the other hand, cholesterol levels did not show significant changes among groups (1193 ± 310 mg/dL in siCD40 treated group, n = 10; 1159 ± 250 mg/dL in siSC group, n = 5; 1387 ± 475 mg/dL in vehicle group, n = 10; p = ns).

In summary, we previously reported the efficacy of the therapeutic use of an anti-CD40-specific short interference RNA (siRNA) to reduce atherosclerotic lesions in a murine model [15]. Here, we show that the kidneys from mice treated with the siCD40 showed a significantly higher microcapillar density, as the number of CD31/PECAM1 positive endothelial cells per section, than the control mice. On the other hand, our work has also highlighted unwanted side-effects of the siRNA therapy which affected to the renal inflammatory homeostasis (increased macrophage infiltration and NF-κB activation) and to the renal function (increased serum creatinine).

## Discussion

Our group is interested in the cardiovascular complications of CKD patients. We previously demonstrated that treating Apolipoprotein E-deficient (ApoE^−/−^) mice with a siRNA against the costimulatory receptor CD40 (siCD40) ameliorated ATH progression, and here we have deepened on the physiological mechanisms involved in the siCD40 action in the context of a functional “aorta-kidney axis”. We have shown that the siCD40 treatment impacted on the renal microcapillar density and resulted in an activation of the innate immune response in mice treated for 16 weeks, over the values found in the control groups treated with vehicle (PBS) or with a scrambled-sequence siRNA (siSC) also during 16 weeks.

The Atherosclerosis-prone, C57BL/6 J ApoE^−/−^ mice model is characterized by a poor liver lipoprotein clearance of diet-derived chylomicrons and liver-derived VLDL remnants with accumulation of cholesterol ester-enriched particles in blood [20]. Hypercholesterolemia (HC) can impair both the function and structure of large and small vessels, thus damaging target organs, as well as to increase renal oxidative stress [2]. In our previous study in ApoE^−/−^ mice, progression of ATH was associated with increased local inflammation in plaques from aorta, while systemic CD40 silencing was associated with a reduction in the number of plaques infiltrating macrophages, intimal CD40+ cells and NF-κB expressing cells, but the kidneys from the treated mice could not be analysed [15]. In this extension study, we have observed that renal MC density was higher in mice treated with the siCD40 than in the controls (siSC or Veh groups), suggesting that the degeneration of the vascular walls associated to a marked increase in total plasma cholesterol could be prevented by silencing CD40. In this sense, it has been proposed that endothelial dysfunction, vascular remodelling and the loss of the renal microvessels played a prominent role in inducing renal injury associated with major cardiovascular risk factor [21], with the CD40/CD40L system contributing to the enhanced microvascular thrombosis associated with models of inflammation and with CD40 deficiency protecting against thrombosis [13].

ApoE^−/−^ mice display renal injuries characterized by glomerular macrophage infiltration with accumulation of foam cells, foci of mesangiolysis, focal intracapilary lipid deposits and foam cell transformation of arterial smooth muscle cells in glomerular hiliar arterioles [22]. In this work, the two groups of siRNA-treated animals, the siCD40 and the siSC group showed a significantly higher infiltration by macrophages, as well as an overactivation of the NF-κB pathway when compared with the group of animals treated with the vehicle only. This increase in inflammatory markers was further associated with a loss of renal function as measured by increased levels of serum creatinine (3.4 ± 3.3 mg/dL in the siCD40 and 4.6 ± 3.0 mg/dL in SC vs. 1.1 ± 0.9 mg/dL in Veh.; *p* = 0.1), suggesting the existence of kidney off-target effects for the siRNA therapy. Interestingly, the siSC-treated group showed higher macrophage infiltration and a peak of NF-κB activation when compared with the siCD40, a fact suggesting a protective effect by the specific siCD40 sequence, likely by interfering the inflammatory cascade.

The existence of unwanted side-effects is hindering the therapeutic potential of siRNAs. RNA Interference (RNAi)-based therapies are slowly permeating the clinical practice, specially to treat undruggable targets [23], although much work has yet to be done regarding their stability, bioavailability, safety and specificity, target validation, tissue specificity, and transfection/transduction efficiency [24, 25] prior to considering them as established therapies for different diseases, from cancer to CVD [26, 27]. Among this “to-do” list, the question of specificity is one of the most important and difficult to resolve, because siRNAs are promiscuous by nature and a single siRNA may target more than one mRNA (even through imperfect base-pairing) to cause off-target silencing [28]. To reduce these unwanted effects a number of strategies have been devised, as reducing the concentration of siRNA used [28], modifying the structure of the siRNA backbone [29], introducing chemical modifications to the seed sequence [30] or introducing sequential information and structural features of the sequences flanking the seed sequence [31]. Furthermore, a number of algorithms [32, 33] and databases [34, 35] have been established to facilitate the development of more specific siRNAs with less off-targets effects.

Interestingly, our control siRNA (siSC), which had the same base composition of the siCD40 but in a random sequence and was designed to highlight sequence-dependent effects, also showed significant off-target effects, such as the infiltration by macrophages and the overactivation of the NF-κB pathway, suggesting a role for the siRNA backbone itself, its modifications or the delivery vehicle, in the generation of the unwanted effects. In this sense, it is known that siRNAs can activate innate immunity (see [36] for a recent review), through the binding of double-stranded RNA by Toll-like Receptors (TLR), a family of innate-recognition receptors that recognize molecular patterns associated with microbial pathogens and activate the NF-κB pathway [37]. Furthermore, several studies have demonstrated that the immune response to siRNA is cell type-dependent, due to the selective expression of TLRs. SiRNAs stimulate monocytes and myeloid dendritic cells, through TLR8, to produce pro-inflammatory cytokines or to activate plasmacytoid dendritic cells, and through TLR7, to produce type 1 interferons [38]. In addition, it has been reported that an unmodified, naked, anti-caspase-3 siRNA is able to pass through the glomerular filtration barrier into the urine [39], aggravating renal graft injury [40] and suggesting a potential mechanism of renal toxicity. On the other hand, although we have herein reported an increase in macrophage infiltration and NF-κB activation in the kidneys of ApoE^−/−^ mice treated with siSC or siCD40 versus mice treated with vehicle, in a previous work using the same siCD40 in the NZB/NZW F1 mice model of lupus nephritis we did not notice any evidence of activation of TLR3, TLR4 or TLR9 [16]. Furthermore, our group has also described that the systemic injection of siCD40 in rat models of renal transplantation did not activate the local innate immune response, as no changes in TLR3 or NF-κB expression were detected [17]. These results suggest that stimulation of the innate immune system by siRNAs might be also dependent of the microenvironment or on the animal genetic background.

Lastly, this study has highlighted unwanted off-target side effects of the otherwise efficient anti-CD40 siRNA therapy, and more strikingly of the scrambled sequence siRNA used as control in the animal experiments. Off-target side effects are one of the major obstacles for siRNA therapy [38], since it has been demonstrated that siRNAs can even induce significant changes in cell viability in a target-independent fashion [41]. In this context, our results call for a more careful consideration of the impact of siRNAs on the innate immune system prior to establish highly efficient siRNA therapies. Clearly, more work on the range of siRNA dosages administrated, on the vehicles used for administration as well as in the administration frequency will be needed to reduce the off-target effects of siRNA therapies and to guarantee their efficacy and safety. On the other hand, the discovery of the off-target effects of the siSC control is, certainly, a flaw on the preclinical use of specific siRNAs in experimental animal models since this is a very popular control, although it remains to be studied whether the effects here described were kidney-specific or part of a more systemic effect and some doubts were previously casted on its utility as experimental control [42].

## Conclusions

ATH was associated with a reduction in renal microcapillary density, an effect reversed by the immunomodulatory effect of anti-CD40 siRNA treatment. However, careful consideration of the impact of siRNA loads on the innate immune system as well as in the delivery is required to reduce the unwanted off-target side effects and to guarantee the safety of siRNA therapies.

## Methods

### Animal models

In this work we used kidney tissue samples from 25 female ApoE^−/−^ mice (Jackson Laboratory, Charles River, Wilmington, MA, USA) from a previous study [15]. Briefly, 8 weeks old mice were treated twice weekly with an intraperitoneal administration of 50 μg of siRNA against CD40 (siCD40), of a scrambled siRNA (SC) as control, or with phosphate buffered saline as vehicle (Veh) for 16 weeks. Mice were euthanized by isoflurane anaesthesia after 16 weeks of treatment (SC/24w, *n* = 5; siCD40/24w, *n* = 10; and Veh, n = 10). To accelerate atherosclerosis, mice were fed with a western diet that contained 0.2% cholesterol, and provided 42% calories as fat (TD.88137; Harlan-Tekland, Madison, WI, USA). All animal studies were in accordance with EU guidelines on animal care and the protocols were approved by the ethics committee for animal research of the University of Barcelona-HUB.

### Materials, siRNAs and antibodies

The sequences, preparation and use of the small interference RNAs (siRNAs) used in this work have been already described [16, 43]. The commercial synthetic siRNAs (siCD40 sense 5′-GUGUGUUACGUGCAGUGACUU, siCD40 antisense 5′-AAGUCACUGCACGUAACACACUG, siSC sense 5′-ACUACAAGACUCGUGACCAUU, siSC antisense 5′-UGGUCACGAGUCUUGUAGUUU, all from Mycrosynth, Switzerland) included stabilizing phosphorothioate backbone and 2′-O-methyl sugar modifications, as well as a cholesterol molecule added at the 3′ end of the sense strand with a pyrolidine linker to facilitate uptake and internalization.

For the immunochemical work, we used a rat monoclonal antibody against F4/80 (Hycult biotech, Uden, NL), a rabbit polyclonal anti-NF-κB-p65 (anti-phospho-S536, Ab86299, Abcam, Cambridge, UK), and a goat polyclonal anti-CD31/PECAM-1 (platelet-endothelial cell adhesion molecule-1) antibody (M-20, sc-1506, Santa Cruz Biotechnology, Santa Cruz, CA). As a secondary antibody we used a goat anti-rat IgG2 (Novus Biologicals, Littleton, CO), a biotinylated horse anti-goat IgG (BA-9500, Vector, Burlingame, CA) or a Vectastain Elite ABC kit for rabbit antibodies (PK-4001-NB, Vector Laboratory, Burlingame, CA).

### Immunohistochemistry

Here, we have studied the microcirculation and inflammation in the kidneys of the ApoE^−/−^ model mice by using standard procedures of Immunohistochemistry. Formalin-fixed, paraffin-embedded kidneys were sliced at 3 μm, stained with the rat monoclonal against F4/80, or with the rabbit polyclonal anti-NF-κB-p65 (Abcam, Cambridge, UK) and counterstained with haematoxylin to make nuclei evident. Human parotid glands or ganglions were used as positive controls. Isotype controls were performed by staining uniquely with antibody buffer supplemented with irrelevant immunoglobulines of the same isotype, species and concentration as the primary antibodies (ThermoFisher, Rockford, IL USA). Negative controls were performed by omitting primary antibodies in the staining. Macrophage infiltration was assessed by counting the total number of F4/80+ cells by field in a total of 5 kidney sections, while activated endothelial cells were detected after NF-κB-p65 nuclear staining. Positively stained cells were counted at × 200 magnifications by capturing images directly from a RGB camera (ProgRes CF scan) attached to a light microscope (NIKON E800). Micro vessel density was assessed by using a systematic random sampling method on microscope kidney sections previously stained for the pan-endothelial marker [44]. We selected five regions of interest (ROIs), on each whole-slide image, on which we measured the density of peritubular vessels per 200x area in tissue sections. We applied digital tools to automate image quantification with ImageJ v1.48. Results were expressed as the proportion of vessel area density per kidney section [44].

### Statistical analysis

Macrophage infiltration and endothelial cells with nuclear NF-κB were measured as the mean ± standard deviation. Differences in cell infiltration and vessel area density between in mice treated with siCD40, scrambled or vehicle were compared by the Kruskal-Wallis test. A *p* < 0.05 value was considered statistically significant. *P* values were corrected for the number of variables compared according to the Bonferroni method. Statistical analysis was performed using the SPSS 20.0 software (SPSS Inc. Chicago, IL).

## Data Availability

The datasets herein used and analyzed are available from the corresponding author on reasonable request.

## Abbreviations

ATH: Atherosclerosis
CKD: Chronic Kidney Disease
HC: hypercholesterolemia
MC: microcirculation
MV: microvascular network
NF-κB: Nuclear Factor κB
NO: Nitric Oxide
PECAM-1: platelet-endothelial cell adhesion molecule-1
siCD40: anti-CD40 siRNA
siRNA: small interfering RNA
siSC: sequence scrambled siRNA
TLR: Toll-like Receptors

## References

[1] Hansson GK, Libby P (2006). The immune response in atherosclerosis: a double-edged sword. Nat Rev Immunol.

[2] Chade AR, Krier JD, Galili O, Lerman A, Lerman LO (2007). Role of renal cortical neovascularization in experimental hypercholesterolemia. Hypertension.

[3] Chen CH, Henry PD (1997). Atherosclerosis as a microvascular disease: impaired angiogenesis mediated by suppressed basic fibroblast growth factor expression. Proc Assoc Am Physicians.

[4] Mattson DL (2003). Importance of the renal medullary circulation in the control of sodium excretion and blood pressure. Am J Physiol Regul Integr Comp Physiol.

[5] Schmieder RE, Hilgers KF, Schlaich MP, Schmidt BM (2007). Renin-angiotensin system and cardiovascular risk. Lancet.

[6] Xiao L, Liu Y, Wang N (2014). New paradigms in inflammatory signaling in vascular endothelial cells. Am J Physiol Heart Circ Physiol.

[7] Evans RG, Gardiner BS, Smith DW, O'Connor PM (2008). Intrarenal oxygenation: unique challenges and the biophysical basis of homeostasis. Am J Physiol Renal Physiol.

[8] Rabelink TJ, de Boer HC, van Zonneveld AJ (2010). Endothelial activation and circulating markers of endothelial activation in kidney disease. Nat Rev Nephrol.

[9] London GM, Guérin AP, Marchais SJ, Métivier F, Pannier B, Adda H (2003). Arterial media calcification in end-stage renal disease: impact on all-cause and cardiovascular mortality. Nephrol Dial Transplant.

[10] Zheng L, Sinniah R, Hsu SI (2008). Pathogenic role of NF-kappaB activation in tubulointerstitial inflammatory lesions in human lupus nephritis. J Histochem Cytochem.

[11] Elgueta R, Benson MJ, de Vries VC, Wasiuk A, Guo Y, Noelle RJ (2009). Molecular mechanism and function of CD40/CD40L engagement in the immune system. Immunol Rev.

[12] Vowinkel T, Wood KC, Stokes KY, Russell J, Krieglstein CF, Granger DN (2006). Differential expression and regulation of murine CD40 in regional vascular beds. Am J Physiol Heart Circ Physiol.

[13] Gavins FN, Li G, Russell J, Perretti M, Granger DN (2011). Microvascular thrombosis and CD40/CD40L signaling. J Thromb Haemost.

[14] Lutgens E, Lievens D, Beckers L, Wijnands E, Soehnlein O, Zernecke A, Seijkens T, Engel D, Cleutjens J, Keller AM, Naik SH, Boon L, Oufella HA, Mallat Z, Ahonen CL, Noelle RJ, de Winther MP, Daemen MJ, Biessen EA, Weber C (2010). Deficient CD40-TRAF6 signaling in leukocytes prevents atherosclerosis by skewing the immune response toward an antiinflammatory profile. J Exp Med.

[15] Hueso M, De Ramon L, Navarro E, Ripoll E, Cruzado JM, Grinyo JM, Torras J (2016). Silencing of CD40 in vivo reduces progression of experimental atherogenesis through an NF-κB/miR-125b axis and reveals new potential mediators in the pathogenesis of atherosclerosis. Atherosclerosis.

[16] Ripoll È, Merino A, Herrero-Fresneda I, Aran JM, Goma M, Bolaños N, de Ramon L, Bestard O, Cruzado JM, Grinyó JM, Torras J (2013). CD40 gene silencing reduces the progression of experimental lupus nephritis modulating local milieu and systemic mechanisms. PLoS One.

[17] de Ramon L, Ripoll E, Merino A, Lúcia M, Aran JM, Pérez-Rentero S, Lloberas N, Cruzado JM, Grinyó JM, Torras J (2015). CD154-CD40 T-cell co-stimulation pathway is a key mechanism in kidney ischemia-reperfusion injury. Kidney Int.

[18] Sledz CA, Holko M, de Veer MJ, Silverman RH, Williams BR (2003). Activation of the interferon system by short-interfering RNAs. Nat Cell Biol.

[19] Robbins M, Judge A, MacLachlan I (2009). siRNA and innate immunity. Oligonucleotides.

[20] Pendse AA, Arbones-Mainar JM, Johnson LA, Altenburg MK, Maeda N (2009). Apolipoprotein E knock-out and knock-in mice: atherosclerosis, metabolic syndrome, and beyond. J Lipid Res.

[21] Maric-Bilkan C, Flynn ER, Chade AR (2012). Microvascular disease precedes the decline in renal function in the streptozotocin-induced diabetic rat. Am J Physiol Renal Physiol.

[22] Wen M, Segerer S, Dantas M, Brown PA, Hudkins KL, Goodpaster T, Kirk E, LeBoeuf RC, Alpers CE (2002). Renal injury in apolipoprotein E-deficient mice. Lab Investig.

[23] Bobbin ML, Rossi JJ (2016). RNA interference (RNAi)-based therapeutics: delivering on the promise?. Annu Rev Pharmacol Toxicol.

[24] Ozcan G, Ozpolat B, Coleman RL, Sood AK, Lopez-Berestein G (2015). Preclinical and clinical development of siRNA-based therapeutics. Adv Drug Deliv Rev.

[25] Pradhan-Nabzdyk L, Huang C, LoGerfo FW, Nabzdyk CS (2014). Current siRNA targets inatherosclerosis and aortic aneurysm. Discov Med.

[26] Laina A, Gatsiou A, Georgiopoulos G, Stamatelopoulos K, Stellos K (2018). RNA therapeutics in cardiovascular precision medicine. Front Physiol.

[27] Gupta A, Ahmad A, Dar AI, Khan R (2018). Synthetic lethality: from research to precision cancer nanomedicine. Curr Cancer Drug Targets.

[28] Caffrey DR, Zhao J, Song Z, Schaffer ME, Haney SA, Subramanian RR, Seymour AB, Hughes JD (2011). siRNA off-target effects can be reduced at concentrations that match their individual potency. PLoS One.

[29] Dua P, Yoo JW, Kim S, Lee DK (2011). Modified siRNA structure with a single nucleotide bulge overcomes conventional siRNA-mediated off-target silencing. Mol Ther.

[30] Iribe H, Miyamoto K, Takahashi T, Kobayashi Y, Leo J, Aida M, Ui-Tei K (2017). Chemical modification of the siRNA seed region suppresses off-target effects by steric hindrance to base-pairing with targets. ACS Omega.

[31] Kamola PJ, Nakano Y, Takahashi T, Wilson PA, Ui-Tei K (2015). The siRNA non-seed region and its target sequences are auxiliary determinants of off-target effects. PLoS Comput Biol.

[32] Wang X, Wang X, Varma RK, Beauchamp L, Magdaleno S, Sendera TJ (2009). Selection of hyperfunctional siRNAs with improved potency and specificity. Nucleic Acids Res.

[33] Rasmussen SH, Jacobsen A, Krogh A (2013). cWords – systematic microRNA regulatory motif discovery from mRNA expression data. Silence.

[34] Das S, Ghosal S, Chakrabarti J, Kozak K (2013). SeedSeq: off-target transcriptome database. Biomed Res Int.

[35] Park J, Ahn SH, Cho KM, Gu D, Jang ES, Chi SW (2018). siAbasic: a comprehensive database for potent siRNA-6Ø sequences without off-target effects. Database (Oxford).

[36] Mansoori B, Mohammadi A, Shir Jang S, Baradaran B (2016). Mechanisms of immune system activation in mammalians by small interfering RNA (siRNA). Artif Cells Nanomed Biotechnol.

[37] Alexopoulou L, Holt AC, Medzhitov R, Flavell RA (2001). Recognition of double-stranded RNA and activation of NF-kappaB by Toll-like receptor 3. Nature.

[38] Yang C, Zhang C, Zhao Z, Zhu T, Yang B (2015). Fighting against kidney diseases with small interfering RNA: opportunities and challenges. J Transl Med.

[39] Kanasty R, Dorkin JR, Vegas A, Anderson D (2013). Delivery materials for siRNA therapeutics. Nat Mater.

[40] Yang C, Jia Y, Zhao T, Xue Y, Zhao Z, Zhang J, Wang J, Wang X, Qiu Y, Lin M, Zhu D, Qi G, Tang Q, Rong R, Xu M, Ni S, Lai B, Nicholson ML, Zhu T, Yang B (2013). Naked caspase 3 small interfering RNA is effective in cold preservation but not in autotransplantation of porcine kidneys. J Surg Res.

[41] Fedorov Y, Anderson EM, Birmingham A, Reynolds A, Karpilow J, Robinson K, Leake D, Marshall WS, Khvorova A (2006). Off-target effects by siRNA can induce toxic phenotype. RNA.

[42] Buehler E, Chen YC, Martin S (2012). C911: a bench-level control for sequence specific siRNA off-target effects. PLoS One.

[43] Ripoll E, Pluvinet R, Torras J, Olivar R, Vidal A, Franquesa M, Cassis L, Cruzado JM, Bestard O, Grinyó JM, Aran JM, Herrero-Fresneda I (2011). In vivo therapeutic efficacy of intra-renal CD40 silencing in a model of humoral acute rejection. Gene Ther.

[44] Marien KM, Croons V, Waumans Y, Sluydts E, De Schepper S, Andries L, Waelput W, Fransen E, Vermeulen PB, Kockx MM, De Meyer GR (2016). Development and validation of a histological method to measure microvessel density in whole-slide images of cancer tissue. PLoS One.

